# On the molecular and morphological evolution of continental and insular *Cryptorchestia* species, with an additional description of *C.garbinii* (Talitridae)

**DOI:** 10.3897/zookeys.783.26179

**Published:** 2018-09-03

**Authors:** Domenico Davolos, Elvira De Matthaeis, Leonardo Latella, Marco Tarocco, Murat Özbek, Ronald Vonk

**Affiliations:** 1 INAIL, Research, Certification, Verification Area, Department of Technological Innovations and Safety of Plants, Products and Anthropic Settlements (DIT), Rome, Italy Department of Technological Innovations and Safety of Plants, Products and Anthropic Settlements (DIT) Rome Italy; 2 Department of Biology and Biotechnology ‘Charles Darwin’, Sapienza University of Rome, Viale dell’Università, 32, - 00185 Rome, Italy Sapienza University of Rome Rome Italy; 3 Museo Civico di Storia Naturale of Verona, Lungadige Porta Vittoria 9 – 37129, Verona, Italy Museo Civico di Storia Naturale of Verona Verona Italy; 4 Ege University, Faculty of Fisheries, 35100 Bornova-Izmir, Turkey Ege University Bornova-Izmir Turkey; 5 Naturalis Biodiversity Center, P.O. Box 9517, 2300 RA Leiden, The Netherlands Naturalis Biodiversity Center Leiden Netherlands; 6 Institute for Biodiversity and Ecosystem Dynamics, University of Amsterdam, Science Park 904, Amsterdam 1098 XH, The Netherlands University of Amsterdam Amsterdam Netherlands

**Keywords:** Bayesian analysis, biogeography, northwest Turkey, phylogeny, Talitridae, taxonomy

## Abstract

Semi-terrestrial talitrid amphipods of the genus *Cryptorchestia* (sensu Lowry and Fanini 2013) associated with freshwater-soaked leaf litter were known to occur in inland lakes of Turkey and at the shores of the Black Sea. Before 2013 they had been reported as *Orchestiacavimana* and later as *Cryptorchestiacavimana*. In our phylogenetic tree, inferred from a mitochondrial and nuclear gene dataset (cytochrome oxidase I (COI), and histone H3 (H3), respectively), we show that these Turkish populations belong to *Cryptochestiagarbinii*, a common and widespread continental species, which is closely related to *C.cavimana* (endemic to Cyprus) and *C.ruffoi* (endemic to Rhodes). For the Turkish and European populations of *C.garbinii*, we found low levels of both genetic differentiation and morphological variation, and an age-related size variability (increasing at each moult) of the small lobe in the male gnathopod I merus, the main taxonomically diagnostic character for *Cryptorchestia*. A mainland (*C.garbinii*) versus insular isolation and in situ speciation (*C.cavimana*, and *C.ruffoi*) in the two east Mediterranean islands of Cyprus and Rhodes is discussed in relation to terrestrial *Cryptorchestia* species endemic to North East Atlantic volcanic islands (Azores, Canary Islands, and Madeira). The incorporation of five Mediterranean and Atlantic *Orchestia* species in the Bayesian analysis of the two genes (COI, and H3) indicated that both genera *Orchestia* and *Cryptorchestia* are not monophyletic.

## Introduction

The genus *Cryptorchestia* Lowry & Fanini, 2013 was erected to accommodate semi-terrestrial and terrestrial species, associated with freshwater, set apart from the marine, supralittoral beachhoppers in the genus *Orchestia* Leach, 1814. Up to this date, *Cryptorchestia* contains eleven species (Horton et al. 2018) and is mainly distributed over several North East Atlantic islands (Azores, Canary Islands, Madeira), Europe, and over the Eastern Mediterranean, including the islands of Rhodes and Cyprus (Davolos et al. 2017).

Semi-terrestrial talitrid amphipods of the genus *Cryptorchestia* associated with freshwater-soaked leaf litter were known to occur at the shores of inland lakes of Turkey and also of the Black Sea. Before 2013 they had been reported as *Orchestiacavimana* Heller, 1865 and after that as *Cryptorchestiacavimana* (Heller, 1865), without considering that *Cryptorchestiacavimana* is endemic to Cyprus (Ruffo et al. 2014, Davolos et al. 2017). Özbek et al. (2004) listed the Malacostraca species of the Iznik and Uluabat lakes and recorded *O.cavimana* (here identified as *C.garbinii* Ruffo, Tarocco and Latella, 2014) from those lakes. Later, Özbek (2011) summarized the distribution of Ponto-Caspian amphipod species in Turkey and mentioned the presence of *O.cavimana* (*C.garbinii*, present study) in Sapanca, Iznik, and Uluabat lakes in the Sakarya and Bursa provinces. A southern record of similar looking specimens was reported from Eğirdir Lake, which is located in the Lake District Region of Turkey (Özbek and Ustaoğlu 2005). Recently, Özbek et al. (2017) reported *C.cavimana* (probably *C.garbinii*, see below) from a creek near Silivri, a town bordering the northern coast of the Sea of Marmara.

As part of the studies on the taxonomy and distribution of the genus *Cryptorchestia* in countries bordering the Mediterranean Sea, talitrid specimens were collected at the shore in a river near Kiyiköy, and in Lake Iznik and Lake Sapanca. These localities are in the Marmara region of Northwest Turkey. Our phylogenetic analysis based on mitochondrial (mt) and nuclear gene sequences confirmed that these Turkish populations belong to *C.garbinii*. Here, we also used morphological comparative methods for the Turkish populations in relation to European conspecific populations, and provided an additional description of *C.garbinii* to report on intraspecific variation.

In order to investigate evolutionary patterns, our study compared the continental *C.garbinii* in relation to the insular *C.cavimana* (Cyprus), *C.ruffoi* (Rhodes), and *Cryptorchestia* species from NE Atlantic islands (Azores, Canary Islands, Madeira). Finally, our molecular framework, obtained by means of Bayesian analysis that included five Mediterranean/Atlantic *Orchestia* species, indicated that both the genera *Orchestia* and *Cryptorchestia* are not monophyletic.

## Materials and methods

### Population sampling

**Figure 1.**
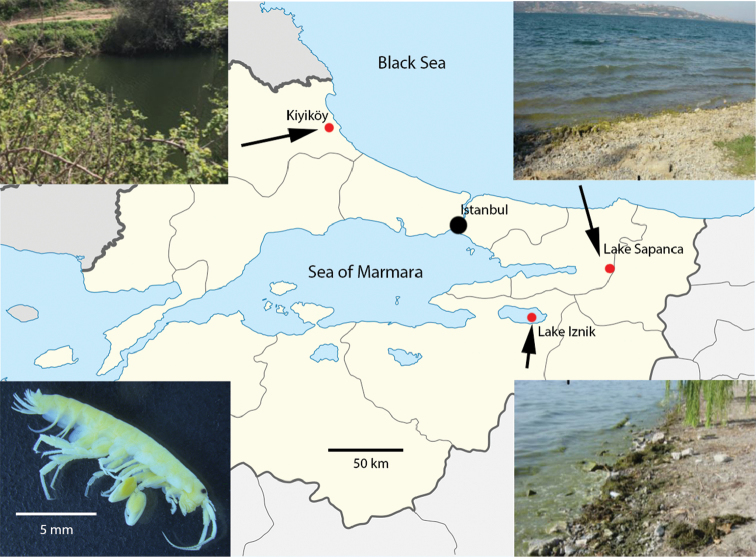
Occurrence of *Cryptorchestiagarbinii* in the Marmara region of Turkey and the habitats (insets) where specimens were collected. The 15 mm male (inset) of *C.garbinii* was found in Lake Sapanca.

**Figure 2.**
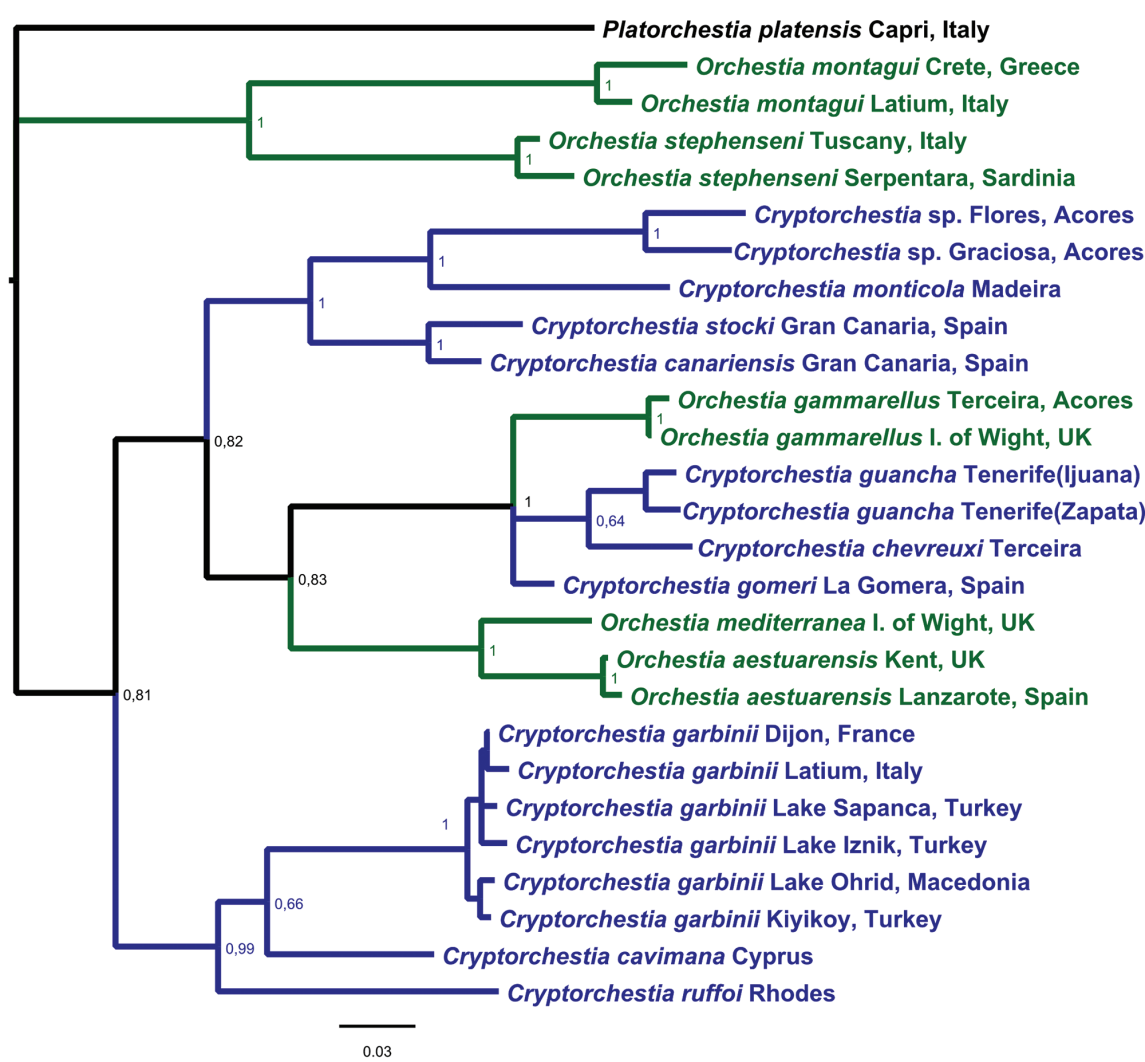
Molecular phylogeny by Bayesian method obtained in a combined analysis using mitochondrial cytochrome oxidase I (COI) gene region (363 bp), and H3 histone (H3) gene fragment (330 bp) sequences (a total of 693 positions in the final dataset) from *Cryptorchestia* and *Orchestia* species reported in Table 2. *Platorchestiaplatensis* was used in this study as an outgroup species. Marked in blue: *Cryptorchestia* species; marked in green: *Orchestia* species. Numbers at nodes correspond to Bayesian posterior probability (PP) support values; PP values greater than 0.5 are labelled. The GenBank accession numbers of the DNA sequences from the COI and the histone H3 genes used in this study are reported in Table 2.

The specimens analysed were collected from three localities in the Marmara region (Fig. 1). A total of 23 specimens were examined for an additional description and measurements of Turkish *C.garbinii* populations. Most specimens were preserved in 70% ethanol, and some were mounted on glass slides in Faure’s medium for morphological observation, others were immediately preserved in 96% ethanol in the field. The photo of a male (Fig. 2) was obtained with a stereo microscope Leica M 165c, mounted with a Leica DFC450 camera at the Museo Civico di Storia Naturale of Verona. The material is deposited in the Museo Civico di Storia Naturale (MSNVR), Verona, Italy, and the Naturalis Biodiversity Center (RMNH), Leiden, The Netherlands.

### DNA isolation, PCR amplification, and DNA sequencing

Specimens of *C.garbinii* from Lake Iznik (Bursa Province), Lake Sapanca (Sakarya province), and a stream near Kiyiköy (Kirklareli province) (Fig. 1) were collected in the end of August 2003 and stored in 96% ethanol. Genomic DNA was extracted from pereopods or whole organisms using QIAamp DNA Mini kit (QIAGEN). A PCR product of ca. 400 base pairs (bp) was amplified from the gene encoding the mt COI (Davolos et al. 2017). The PCR-mediated reaction was performed using the primers BI-COI and SUBIR cited in Davolos and Maclean (2005). The PCR amplification conditions were cited in Davolos et al. (2017). The amplified fragments were checked by electrophoresis in 1% agarose gels and then used as templates for cycle sequencing reactions (BigDye chemistry) followed by DNA sequencing using BI-COI and SUBIR primers. In addition, a fragment of ca. 350 bp of the gene encoding the nuclear histone H3 (H3), was PCR amplified using the primers H3Of and H3Or cited in Davolos and Pietrangeli (2014). The PCR cycling parameters were cited in Davolos et al. (2017). The PCR products were verified and then sequenced using H3Of and H3Or primers, as above described.

The novel annotated sequences from the mt COI and the nuclear H3 genes from *C.garbinii* of this study have been submitted to the GenBank databases at the National Center for Biotechnology Information (NCBI; http://www.ncbi.nlm.nih.gov) (Table 2). Other DNA sequences used for the Bayesian analysis of the present study were already deposited at NCBI by Davolos and reported in Davolos et al. (2017). In more details, the European populations of *C.garbinii* are represented by samples from France (Dijon), and Italy (Latium; Table 2). DNA sequences of *C.cavimana* (Davolos et al. 2018) and *C.ruffoi* Latella & Vonk, 2017 are cited in Davolos et al. (2017). We added DNA sequences from *Cryptorchestia* species of the northeast Atlantic volcanic islands examined in our previous study (*C.canariensis* (Dahl, 1950), *C.gomeri* (Stock, 1989), *C.guancha* (Stock & Boxshall, 1989), and *C.stocki* (Ruffo, 1990) from the Canary Islands, *C.monticola* (Stock & Abreu, 1992) from Madeira, and *C.chevreuxi* (De Guerne, 1887) from Terceira, Azores (Davolos et al. 2017). Moreover, additional unpublished sequence data of undescribed *Cryptorchestia* taxa from the islands of Graciosa and Flores, Azores, were obtained from the work of Davolos and De Matthaeis (2017). We also included sequences from the mt COI and the nuclear H3 genes of five *Orchestia* species, two Mediterranean endemic species, *O.montagui* Audouin, 1826 and *O.stephenseni* Cecchini, 1928, and the Mediterranean/Atlantic *O.gammarellus* (Pallas, 1976), *O.mediterranea*, Costa, 1853, and *O.aestuarensis* Wildish, 1987. These sequences were obtained from a former project of Davolos and De Matthaeis and are available from GenBank (see Table 2 for accession numbers). For the outgroup we used DNA sequences of *Platorchestiaplatensis* (Krøyer, 1845) from the Island of Capri, Italy (see Table 2).

### Molecular phylogeny

A Bayesian phylogenetic inference analysis was carried out using the MrBayes v.3.2.6 software package (Ronquist et al. 2012). We concatenated the two genes into a single alignment before Bayesian methods were conducted. The General Time Reversible plus gamma distributed rate variation among sites (G), and proportion of invariable sites (I) in the dataset, GTR+G+I substitution model, was selected for the two genes COI, and H3. Analyses were run for 10 million generations, with trees sampled every 1000 generations and a burn-in of 25%. Two independent runs with three hot chains and one cold chain were conducted, which were long enough to effectively sample each distribution and to reach convergence (e.g., the standard deviation of split frequencies was 0.004, the potential scale reduction factor (PSRF) was close to 1.0 for all parameters, and the effective sample size (ESS) was high for all the parameters). The first 25 % of trees, which represented the burn–in phase of the analysis, were discarded. The remaining trees were used for calculating posterior probabilities (PP) of recovered branches in the 50 % majority rule consensus tree. Subsequently, the tree was graphically visualized and exported by FigTree v1.4.2 (http://tree.bio.ed.ac.uk/software/figtree/) for further editing with the program Inkscape (http://www.inkscape.org).

## Results

### Molecular phylogenetic analysis

Mitochondrial and nuclear genes (COI, and H3, respectively) of the newly sequenced Turkish specimens of *C.garbinii* and other talitrids for a total of 27 taxa were used for phylogenetic analysis. Concatenation of DNA sequence data produced an alignment of 693 bp.

The results from the Bayesian inference phylogenetic analysis are presented in Fig. 2. In the COI-H3 phylogeny constructed with Mr Bayes, the phylogenetic clusters generally were well supported (PP above 0.8) and some clades were very well supported (PP > 0.95). A first clade grouped with full support (PP of 1) *O.montagui* and *O.stephenseni*, both endemic to Mediterranean marine supralittoral. Moreover, our phylogenetic analysis recovered a monophyletic clade (PP = 0.99) that was formed by the continental species *C.garbinii*, widely distributed from Turkey (present study) to central Europe, and two other freshwater *Cryptorchestia* species, *C.cavimana*, and *C.ruffoi*, which are endemic to the east Mediterranean islands of Cyprus and Rhodes, respectively. Within *C.garbinii*, apparently the European populations from France (Dijon) and Italy (Latium) are more related to the Turkish populations from Lake Iznik and Lake Sapanca, while the Kiyiköy population was genetically more related to the population of Lake Ohrid in Macedonia.

Finally, our phylogenetic analysis recovered a group of talitrid taxa from NE Atlantic area (PP of 0.82), which was divided into two subgroups. A monophyletic subgroup showed very well-supported relationships (PP of 1) for the terrestrial *Cryptorchestia* lineages of *C.canariensis*, and *C.stocki* endemic to Gran Canaria, Canary Islands, *C.monticola* endemic to Madeira, and undescribed *Cryptorchestia* taxa from the islands of Graciosa and Flores, Azores (Davolos and De Matthaeis 2017). The second monophyletic clade (PP of 0.83) was further divided into subclades. One subclade was formed by the terrestrial *Cryptorchestia* lineages of *C.guancha* endemic to Tenerife, Canary Islands, and *C.chevreuxi* from Terceira, Azores, while *C.gomeri* endemic to La Gomera, Canary Islands, and *O.gammarellus* (known to occur from fully marine to freshwater shores) as sister species. The other subclade (PP of 1) was formed by the two morphologically related but genetically different *O.mediterranea* and *O.aestuarensis* (Fig. 2), both species well known to occur even in low salinity conditions.

### Systematics

#### Order Amphipoda Latreille, 1816

##### Suborder Senticaudata Lowry & Myers, 2013

###### Family Talitridae Rafinesque, 1815

####### Genus *Cryptorchestia* Lowry & Fanini, 2013

######## Cryptorchestia garbinii

Taxon classificationAnimaliaAmphipodaTalitridae

Ruffo, Tarocco & Latella, 2014

**Figure 3.**
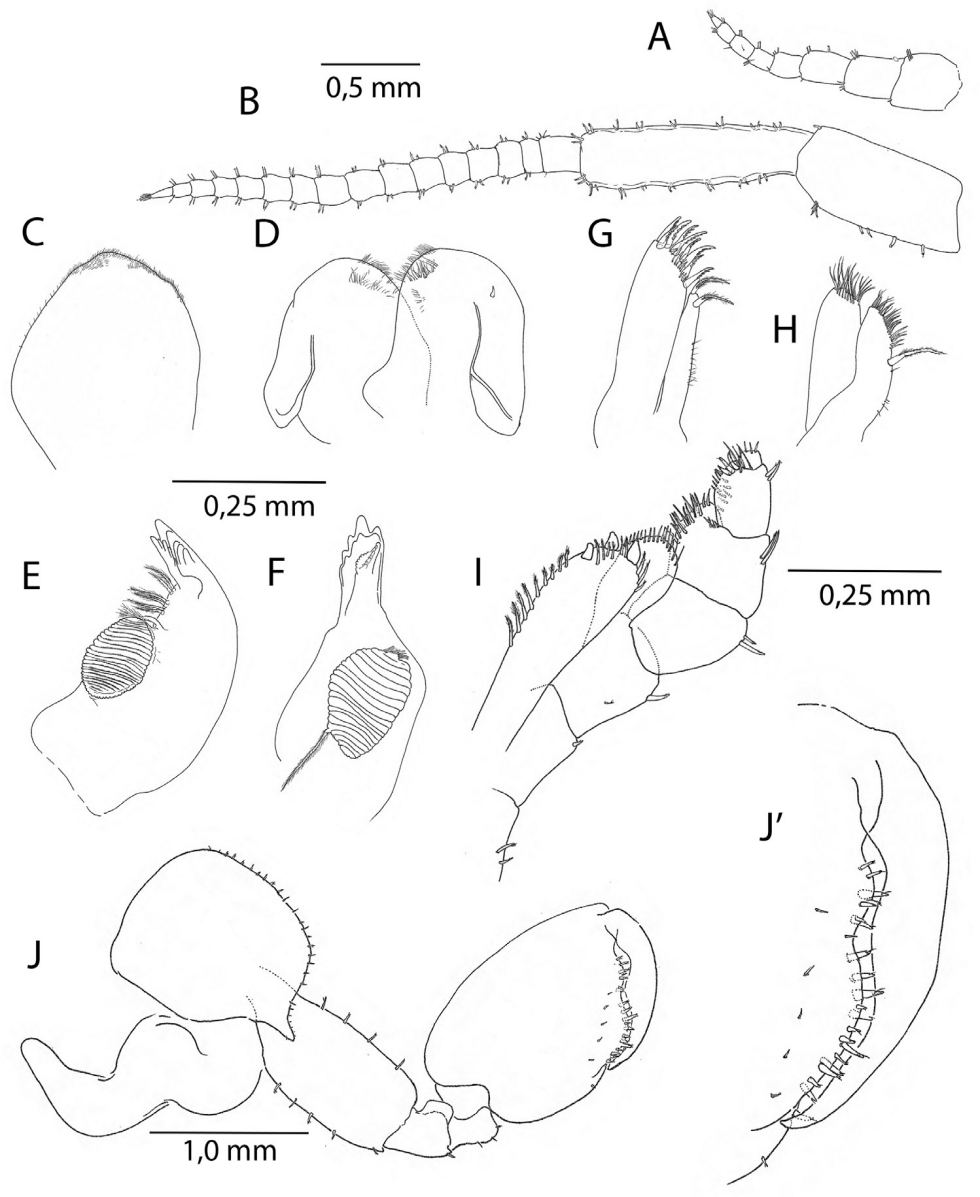
*Cryptorchestiagarbinii*, male 12 mm, Lake Iznik. **A** antenna I **B** antenna 2 **C** upper lip **D** lower lip **E** left mandible **F** right mandible **G** maxilla I **H** maxilla II **I** maxilliped **J** gnathopod II **J**’ gnathopod II, detail.

**Figure 4.**
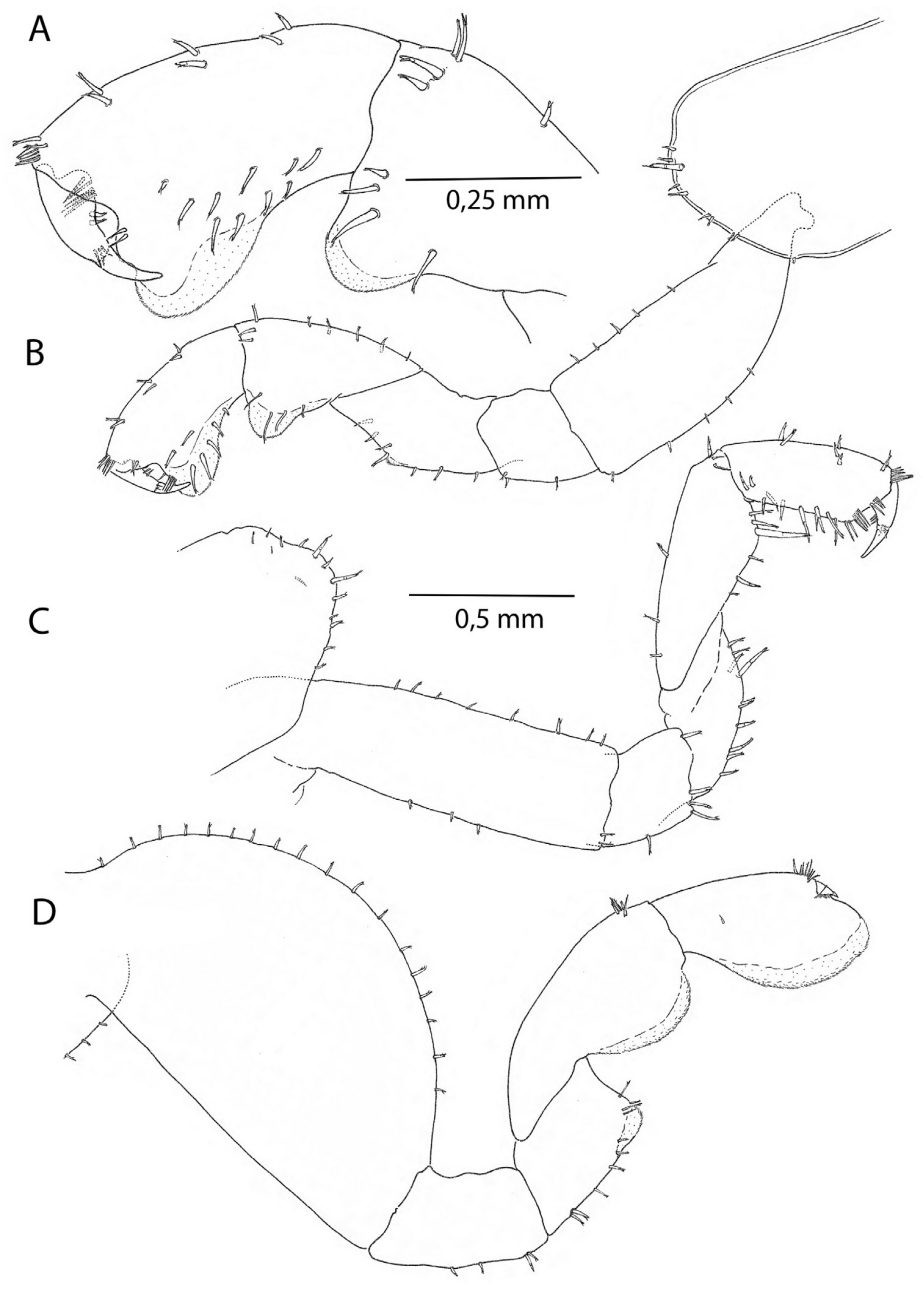
*Cryptorchestiagarbinii*, male 12 mm, Lake Iznik. **A** gnathopod I, outside left **B** gnathopod I, inside right **C** female 13 mm, Lake Iznik, gnathopod I **D** gnathopod II.

**Figure 5.**
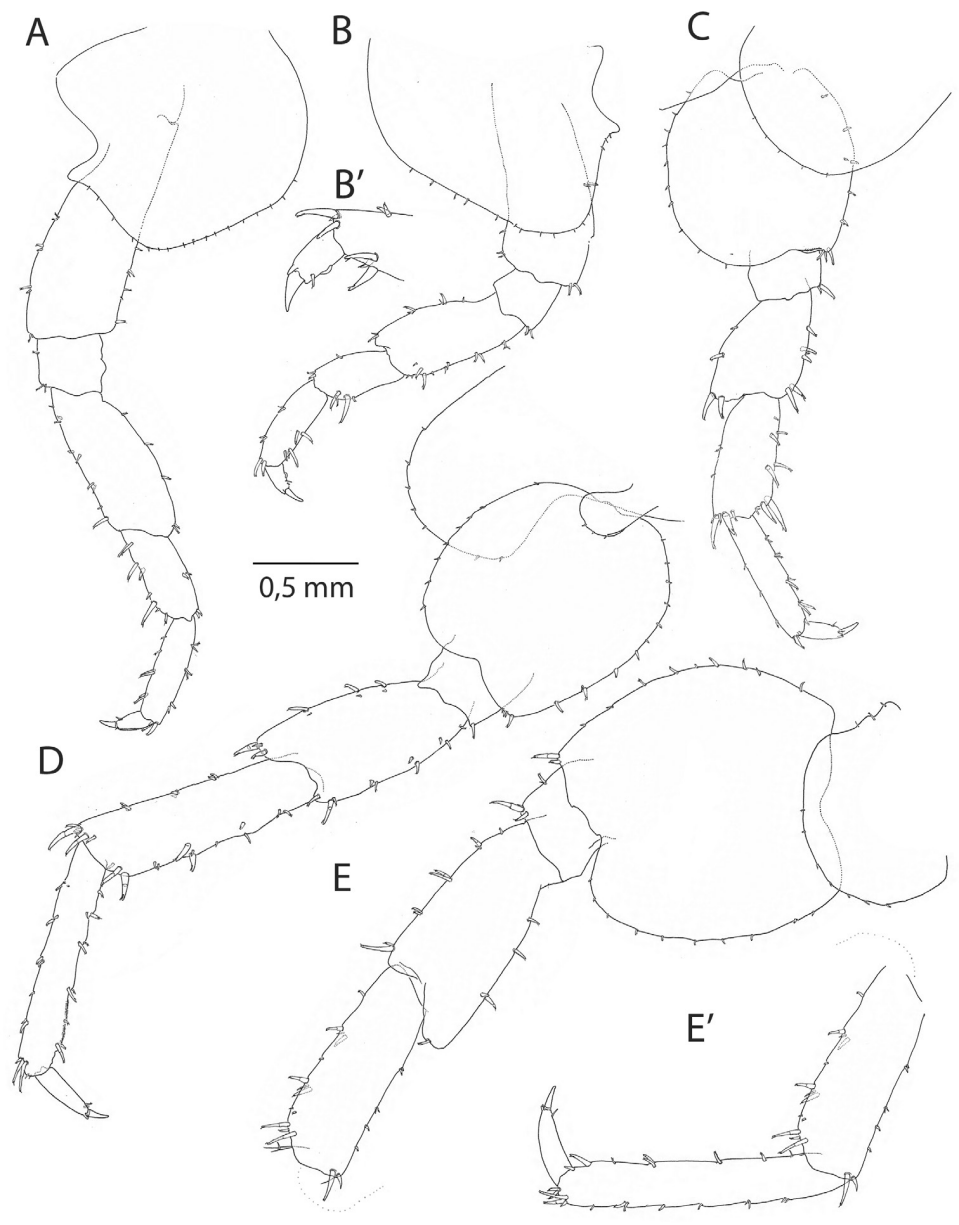
*Cryptorchestiagarbinii*, male 12 mm, Lake Iznik. **A** pereopod III **B** pereopod IV **B**’ pereopod IV, detail dactylus **C** pereopod V **D** pereopod VI **E** pereopod VII **E**’ pereopod VII, carpus, propodus, and dactylus.

**Figure 6.**
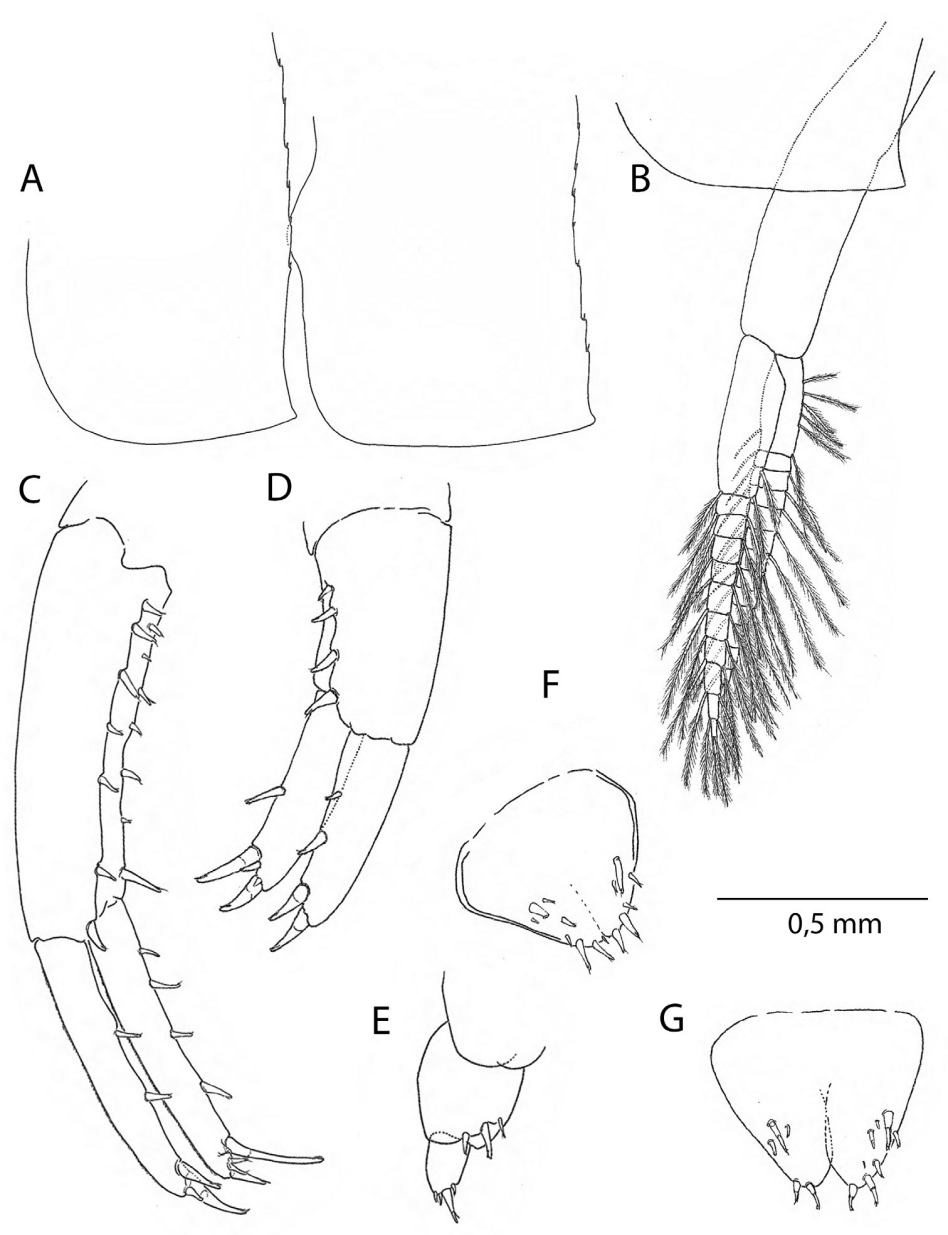
*Cryptorchestiagarbinii*, male 12 mm, Lake Iznik. **A** epimeral plates I and II **B** pleopod II **C** uropod I **D** uropod II **E** uropod III **F** telson **G** female 13 mm, Lake Iznik, telson.

**Figure 7.**
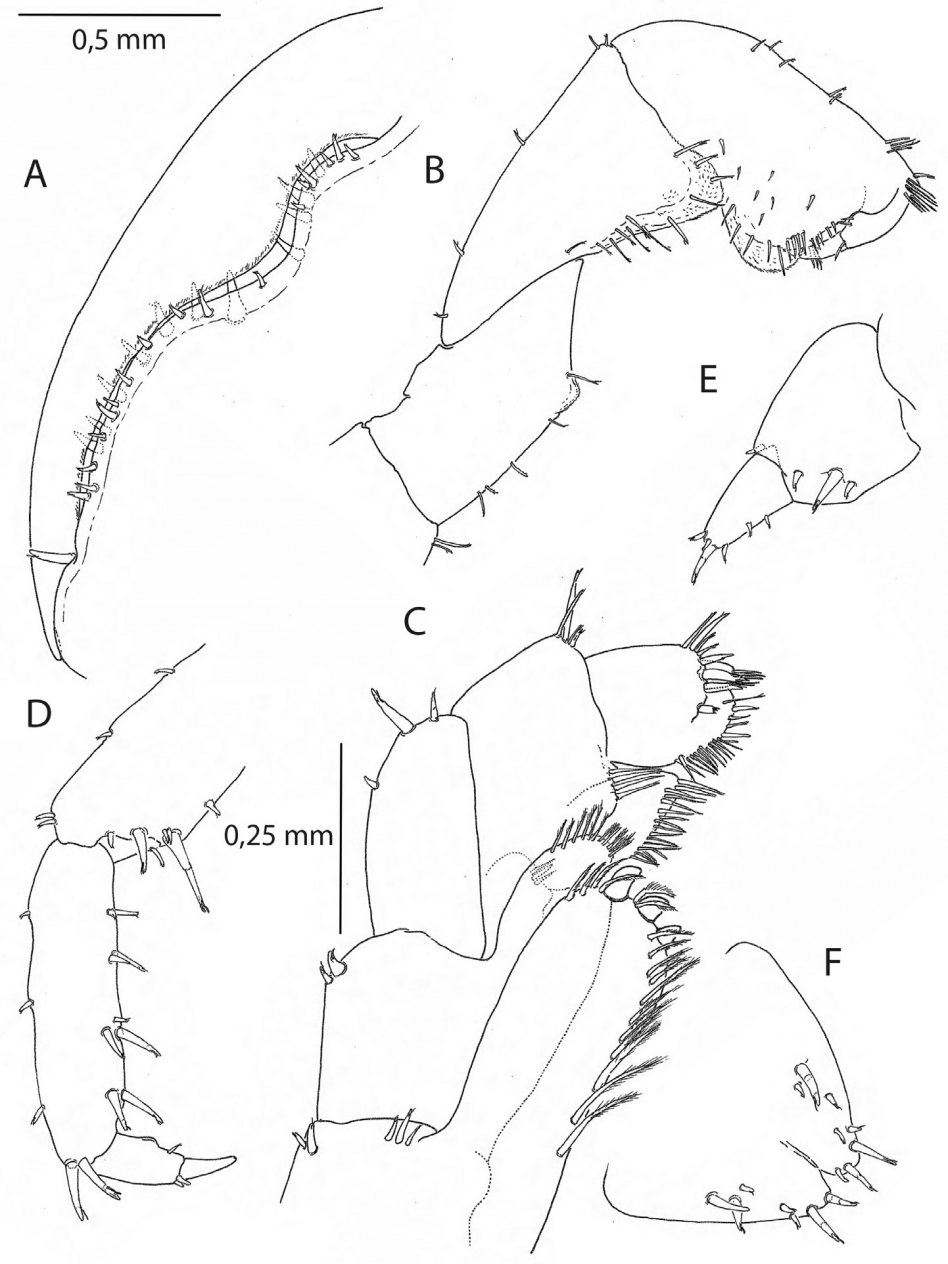
*Cryptorchestiagarbinii*, male 16 mm, Lake Iznik. **A** gnathopod II, palmar margin and dactylus **B** gnathopod I **C** maxilliped **D** pereopod IV, propodus and dactylus **E** uropod III **F** telson.

Figs 3 , 4 , 5 , 6 , 7

######### Localities.

The specimens of *C.garbinii* reported from Turkey were distributed in Lake Iznik (Bursa Province), Lake Sapanca (Sakarya province), and in a stream near Kiyiköy (Kirklareli province) (Fig. 1).

######### Material used for morphological description.

One male of 12 mm, one female of 13 mm from Iznik Lake, coordinates 40°24'20.39"–29°41'52.75", date 28 VIII 2003; one male of 16 mm from Sapanca Lake, 40°42'16.74"–30°11'36.14", 24 VIII 2003. Additional specimens: 6 males, 4 females of Lake Iznik; 6 males, 4 females from Lake Sapanca (Table 1).

**Table 1.** *Cryptorchestiagarbinii* from Turkey. Measurements of body lengths and antennae, showing differences in male and female individuals.

Sex	Total length (mm)	Ant. I length	Ant. II length	Locality
male	15.5	1.5	6.21	Iznik
male	12.56	1.57	5.87	Iznik
male	14.92	1.76	6.84	Iznik
male	16.31	1.55	5.78	Iznik
male	13.44	1.49	6.11	Iznik
male	13.49	1.3	4.5	Iznik
male	15.76	1.79	7.35	Sapanca
male	16.13	1.58	6.46	Sapanca
male	16.27	1.91	7.27	Sapanca
male	13.07	1.37	5.39	Sapanca
male	13.51	1.5	5.8	Sapanca
male	14	1.38	6.47	Sapanca
**Mean**	**14.58**	**1.56**	**6.17**	
female	12.95	1.08	4.2	Iznik
female	12.8	1.2	4.5	Iznik
female	14.13	1.59	5.15	Iznik
female	12.55	1.09	4.39	Iznik
female	12.46	1.16	3.9	Sapanca
female	13.02	1.04	3.66	Sapanca
female	12.87	1.16	3.53	Sapanca
female	14.03	1.41	4.39	Sapanca
**Mean**	**13.10**	**1.22**	**4.21**	

######### Diagnosis.

Talitridae, with antenna I shorter than the combined peduncle segments of antenna II; accessory flagellum absent; mandible without palp, left mandible with 5-dentate lacinia mobilis; maxilla I with nine setae on inner lobe; maxilliped palp article IV reduced; gnathopod I male with a small, partially transparent lobe on merus; pereopod IV with bulged inner margin of dactylus; pereopods 3-7 cuspidactylate; telson variable in shape, longer than wide in holotype from Lake Garda, wider than long in the Turkish populations.

######### Additional description.

Based on adult males with an average length of 14.58 mm (Table 1). **Head.***Antenna I* (Fig. 3A) flagellum with five to seven articles. *Antenna II* (Fig. 3B) little shorter than half of the body length, peduncle article V longer than article IV, flagellum with 16–21 articles. *Labrum* (Fig. 3C) and *labium* (Fig. 3D) with very fine setules on anterior margin. *Mandible* (Fig. 3E, F) left with 5-dentate lacinia mobilis and 5-dentate pars incisiva, right mandible with multi-teethed lacinia mobilis. *Maxilla I* (Fig. 3G) with nine robust and crenelated setae on inner lobe. *Maxilla II* (Fig. 3H) with numerous apical setae, a long and finely pinnate seta on inner margin of inner lobe. *Maxilliped* (Figs 3I, 7C) basal lobe with three blunt teeth on anterior margin, axial margin lined with robust setae armed with setules; palp article IV reduced, but clearly visible.

*Coxae*. Coxal plate I (Fig. 4B) with irregularly placed larger and smaller setae on distal margin. Coxal plates II-IV wider than deep (Figs 3J, 5A, B), plate V elongated, bilobate (Fig. 5C), plates VI and VII smaller (Fig. 5D, E).

**Pereon.***Gnathopod I* male (Figs 4A, B, 7B) sexually dimorphic, subchelate; merus with small, partly transparent lobe on posterior margin; carpus with four or more setae on posterior margin, lobe present; propodus with sinoid palm in the 12 mm, but straight in 16 mm individual, and with a transparent lobe covering almost the entire palmar margin; dactylus as long as anterolateral margin of the propodus. *Gnathopod II* (Figs 3J, 7A), subchelate; propodus oviform, palmar margin with shallow sinus in the anterodistal part; *Pereopods 3–4* (Figs 5A, B, 7D) with pereopod III slightly longer; dactylus in pereopod IV with bulged inner margin. *Pereopod V* (Fig. 5C) with wide basis; merus and carpus with pairs of robust setae on both sides of distal margins. *Pereopods III–VII* cuspidactylate. *Pereopod VI* (Fig. 5D) shorter than pereopod VII; coxa with small inside lobe; basis wide; carpus of same length as propodus. *Pereopod VII* (Fig. 5E) basis wide with distinct, rounded posterodistal lobe; propodus longer than carpus.

**Pleon.***Epimeral plates* (Fig. VIA, B), posterior margins weakly crenulate. *Pleopods I-III* (Fig. 6B) well developed, biramous; rami with slender setae; inner ramus slightly shorter than outer. *Uropod I* (Fig. 6C) with two rows of seven axial setae on peduncle; rami of equal length, with three to four apical setae (of which one on the inner ramus longer than others). *Uropod II* (Fig. 6D) peduncle with one row of four axial setae; rami of equal length, outer ramus with four apical setae of which two small, inner ramus with two apical setae. *Uropod III* (Figs 6E, 7E) peduncle with three to four distolateral setae, ramus with four apical setae of which one long, and two additional lateral setae in 16 mm individual. *Telson* (Figs 6F, G, 7F) wider than long, dorsal midline cleft, five to eight marginal and distal setae per lobe of which two regular on the apex.

Female. Based on adult females with an average length of 13.10 mm (Table 1). *Antenna I* short, 1.22 mm length in average, flagellum with four articles. *Antenna II* long, 4.21 mm in average. *Gnathopod I* (Fig. 4C) subchelate; coxal plate lower margin with irregularly placed prominent setae; basis rectangular with several short setae; carpus with one robust and long seta between several smaller on lower margin; propodus with three bundled groups on the palmar margin; dactylus with unguis longer than palm. *Gnathopod II* (Fig. 4D), basis large, with strongly curved anterolateral margin; merus with lobe or patch with fine structures resembling a rasp surface; carpus and propodus expanded, with lateral lobes, propodus with rounded frontal margin and small claw.

######### Remarks.

The specimens from the Turkish lakes Iznik and Sapanca show some slight morphological differences compared to the Lake Garda (type locality) specimens. We interpret this as intraspecific variation. The lobe on the merus of the first gnathopod is smaller in the 12 mm male (Fig. 4B) than in the 16 mm male, especially when the twist in the carpus-propodus part relative to the merus forces the gnathopod to appear in an angle on a mounting slide. The feature is clearer in SEM photographs where it shows a rugose patch.

The specimens from Turkey were also compared to the geographically more adjacent freshwater species *C.ruffoi* from the island of Rhodes, Greece, to *C.cavimana* from Cyprus, and to *C.kosswigi* (Ruffo, 1949) from the Hatay province in southern Turkey. Differences are: a small lobe on merus of gnathopod I in male (large in *C.ruffoi*, not mentioned as present in *C.cavimana* by Heller (1865) in his German text but vaguely present in the figures); antenna I with 5-7 segmented flagellum (5-6 in *C.garbinii* of Lake Garda, four in *C.ruffoi*, six in *C.cavimana*, six in *C.kosswigi*); pereopods V–VII with wide bases and of short and stubby appearance (less wide in *C.garbinii* of Lake Garda, *C.ruffoi* and *C.cavimana*, broad in *C.kosswigi* but here the other segments are long and slender); gnathopod II female with very broad and rounded basis (less in *C.ruffoi*, not mentioned in *C.cavimana*, but prominently present in the *C.cavimana* that was partly redescribed from the British Isles (Lincoln 1979), and present to a lesser extent in *C.kosswigi)*; pereopod IV with clear bulge on dactylus (not mentioned in *C.garbinii* of Garda lake, not present in *C.ruffoi* and not mentioned in *C.cavimana*, present in *C.kosswigi*). We consider these differences as interspecific variation of morphological features.

## Discussion

Our molecular phylogeny based on mitochondrial and nuclear protein-coding genes helps to understand evolutionary relationships between continental (*C.garbinii*) and insular *Cryptorchestia* species from the East Mediterranean region and from the Canary Islands, Madeira, and Azores (North East Atlantic area) examined in this study and reported in Fig. 2 and Table 2.

The molecular data indicated a low level of genetic differentiation within the mainland Turkish and European populations of *C.garbinii*. It is noteworthy that DNA sequence data from Kiyiköy on the west side of the Bosphorus indicated that this Turkish coastal population of *C.garbinii* was highly related to that from Lake Ohrid, Macedonia, while the DNA sequences from the Turkish lake inhabitants on the east side from Iznik (Bursa Province) and Sapanca (Sakarya province) in the north-west pointed to a strong relationship with those from Europe, here represented by samples from France (Dijon), and Italy (Latium) (Fig. 2). The low intraspecific genetic diversification within *C.garbinii* and its wide geographic distribution (Figs 1–2) both lead to the supposition that dispersal events have occurred relatively recently. The evolution of *C.garbinii* could have been a pattern of recent east-to-west dispersion, with a more recent northward expansion. A more extensive sampling of other representative localities of *C.garbinii* is required to resolve the phylogeography of this species in greater detail.

**Table 2.** The Mediterranean and North-East Atlantic *Cryptorchestia* and *Orchestia* species employed in molecular analysis based on mitochondrial COI gene region (363 bp), and H3 histone gene fragment (330 bp), the sampling locations, and the GenBank accession numbers. *Platorchestiaplatensis* used in this study as outgroup species is also reported. The NA abbreviation stands for ‘not available’.

Species	Sampling locality	GenBank ace. numbers		
		COl	H3	Reference
*Cryptorchestiacanariensis* (Dahl, 1950)	Gran Canaria, Canary Islands, Spain	KY225807	KY225817	Davolos et al. 2017
*Cryptorchestiacavimana* (Heller, 1865)	Troodos Mountains, Cyprus	KY225808	KY225818	Davolos et al. 2017
*Cryptorchestiachevreuxi* (de Guerne, 1887)	Terceira, Azores, Portugal	NA	KY225819	Davolos et al. 2017
*Cryptorchestiagarbinii* Ruffo, Tarocco & Latella, 2014	Lake Ohrid, Macedonia	KY225809	KY225820	Davolos et al. 2017
	Dijon, France	KY225810	KY225821	Davolos et al. 2017
	Latium, Italy	KY225811	KY225822	Davolos et al. 2017
	Lake lznik, Turkey	MH028384	MH028387	present stud
	Lake Sapanca, Turkey	MH028385	MH028388	present study
	Kiyikoy, Turkey	MH028386	MH028389	present study
*Cryptorchestiagomeri* (Stock, 1989)	La Gomera, Canary Islands, Spain	NA	AM748658	Villacorta et al. 2008
*Cryptorchestiaguancha* (Stock & Boxshall, 1989)	Zapata, Tenerife, Canary Islands, Spain	KY225812	KY225823	Davolos et al. 2017
	ljuana, Tenerife, Canary Islands, Spain	KY379013	KY378977	present study
*Cryptorchestiamonticola* (Stock & Abreu, 1992)	Madeira Island, Portugal	KY225813	KY225824	Davolos et al. 2017
*Cryptorchestiaruffoi* Latella & Vonk, 2017	Rhodes Island, Greece	KY225814	KY225825	Davolos et al. 2017
*Cryptorchestiastocki* (Ruffo, 1990)	Gran Canaria, Canary Islands, Spain	KY225815	KY225826	Davolos et al. 2017
*Orchestiaaestuarensis* Wildish, 1987	Kent, England	KY379023	KY378988	present study
	Lanzarote, Canary Islands, Spain	KY379024	KY378989	present study
*Orchestiagammarellus* (Pallas, 1976)	Isle of Wight, England	AY185148	KY378996	Davolos and Maclean 2005; present study
	Gruta das Agulhas, Terceira, Azores, Portugal	MH042533	MH042534	present study
*Orchestiamediterranea* Costa, 1853	Isle of Wight, England	AY185150	KY378997	Davolos and Maclean 2005; present study
*Orchestiamontagui* Audouin, 1826	Latium, Italy	KY379029	KY378999	present study
	Platanias,Crete, Greece	KY379009	KY378973	present study
*Orchestiastephenseni* Cecchini, 1928	Tuscany, Italy	KY379027	KY378992	present study
	Island of Serpentara, Sardinia, Italy	KY379030	KY379000	present study
*Platorchestiaplatensis* (Kr¢yer, 1845)	Island of Capri, Italy	KY225816	KY225827	Davolos et al. 2017

Our molecular data further suppose an insular isolation and in situ speciation in two east Mediterranean islands, namely *C.cavimana* from the island of Cyprus, and *C.ruffoi* from the island of Rhodes in south-eastern Greece. Moreover, our study supports the monophyletic clade formed by *C.garbinii*, *C.cavimana*, and *C.ruffoi* (Fig. 2), all with a clear small lobe on the merus of gnathopod I. Davolos et al. (2017) added to this group *C.kosswigi* from the Turkish coast that clearly has a small lobe on the male gnathopod I merus (currently no DNA sequences available). These species represent *Cryptorchestiasensu stricto* and are generally found in Mediterranean riparian habitats. Therefore, the pattern of evolution within this semi-terrestrial talitrid lineage appears to have been a process with mainland and insular speciation, with the plausible hypothesis of a common ancestor that occurred in freshwater of the east Mediterranean region.

However, our molecular analysis indicates *Cryptorchestia* not monophyletic (Figure 2) and helps to understand various ecological, morphological, and geographic distribution aspects. It is important to note that semi-terrestrial *Cryptorchestia* species from the east Mediterranean region live in riparian habitats, while the terrestrial species from the islands of the north-east Atlantic area live in humid, evergreen broadleaf laurel forest (laurisilva; see Davolos et al. 2017 and reference therein). Moreover, *Cryptorchestia* has been proposed on a single character (Lowry and Fanini 2013), the gnathopod I merus with a very small lobe on the posterior margin (in *Orchestia* there is a palmate lobe only on male carpus and propodus of gnathopod I), of which the presence is variable within the genus. By focusing on the Atlantic terrestrial *Cryptorchestia* species that live in laurisilva, they appear to be phylogenetically and morphologically distinct and can be divided into two groups (Fig. 2). The first group is formed by *C.guancha*, *C.gomeri*, and *C.chevreuxi*, which share superficially a similar morphology on the absence of the small lobe on the merus of gnathopod I, and they have *O.gammarellus* as sister species. A loss of the lobe in these *Cryptorchestia* species from Macaronesia cannot be ruled out, but it is important to note that they share a common ancestor with the clade formed by *O.mediterranea* and *O.aestuarensis*. Therefore, a plausible hypothesis is that their putative marine common ancestor probably had the sexually dimorphic pereopods I (without the small lobe on the merus), II, and VII. The second group is formed by north-east Atlantic volcanic island *Cryptorchestia* species, some of which (*C.stocki*, and undescribed *Cryptorchestia* species from the Azores (Davolos and De Matthaeis 2017)) have a small lobe on the merus of gnathopod I. Therefore, our study suggests that the morphological convergence of this character in unrelated Mediterranean and Atlantic *Cryptorchestia* species is likely (Fig. 2).

It seems reasonable to assume that the Atlantic volcanic archipelagos, with a sequence of island emergence and ageing over millions of years, might have been colonized by talitrid lineages independently and on different times (Davolos and De Matthaeis 2017). However, we are aware that the phylogeny of *Cryptorchestia* species is far away from being resolved. An important phylogenetic gap exists between the Macaronesian *Cryptorchestia* species and putative close continental relatives, probably from southwest Europe/northwest Africa, even if extinction of the ancestral lineages after the colonization of the Macaronesian volcanic islands cannot be ruled out. A Bayesian phylogeny, using multiple calibrations for divergence time estimation that includes a larger dataset of talitrid genera, such as *Palmorchestia* and *Macarorchestia*, from the North East Atlantic area and the Mediterranean region is one of the major scopes of future work.

However, we have described here, through a multi-locus molecular phylogeny approach, three main clades with species classified as *Cryptorchestia*. Our Bayesian phylogeny showed that the genera *Cryptorchestia* and *Orchestia* as described appear polyphyletic within the Talitridae and indicated that using the current *Cryptorchestia* taxonomy is useful for the *Cryptorchestia* s. s., but that a taxonomic revision within this genus is required. Clearly, our studies confirmed the importance of insular speciation in both the northeast Atlantic and east Mediterranean *Cryptorchestia* lineages.

## Supplementary Material

XML Treatment for Cryptorchestia garbinii
